# Shingles With an Unusual Dermatomal Distribution

**DOI:** 10.7759/cureus.116398

**Published:** 2026-09-17

**Authors:** Nishadi P Galganamehestrige, Deepa Chemjong, Jayashekara Acharya, Alexia Mason, Dony Paul

**Affiliations:** 1 Respiratory Medicine, Wye Valley NHS Trust, Hereford, GBR; 2 Diabetes and Endocrinology, Hereford County Hospital, Hereford, GBR; 3 Hospital Medicine, Wye Valley NHS Trust, Hereford, GBR; 4 Research, Wye Valley NHS Trust, Hereford, GBR

**Keywords:** atypical shingles presentation, herpes zoster (shingles), varicella-zoster, varicella zoster reactivation, varicella-zoster virus

## Abstract

Shingles, also known as herpes zoster, is a globally recognised, self-limiting infection caused by the reactivation of the varicella-zoster virus (VZV). This virus causes varicella, otherwise known as chickenpox, which is a well-known acute infection, commonly seen among children. Herpes zoster involves an individual nerve and the corresponding dermatome. This case report is written with the intent of reminding clinicians of the variety of ways in which herpes zoster can present. It is important to consider herpes zoster in the differential diagnosis of a rash, even when it is confined to a less common dermatome.

## Introduction

Varicella (chickenpox) and herpes zoster (shingles) are two separate clinical manifestations of the same virus: varicella-zoster virus (VZV). Shingles is increasingly common among the ageing population. It is caused by the reactivation of VZV, which can remain dormant in the dorsal root and trigeminal ganglia for years [1] following chickenpox infection, usually during childhood. Herpes zoster is not directly contagious, although active shingles rashes can transmit VZV to those who are not immune via direct contact with weeping blisters. This can lead to chickenpox in those who have not been previously exposed to VZV. Herpes zoster (shingles) is caused by the reactivation of dormant VZV. Weakened immune systems and older age can trigger the reactivation of VZV.

As opposed to chickenpox, which causes a widespread rash across the body, herpes zoster typically presents as a unilateral rash affecting one or two contiguous dermatomes on the thorax. It usually does not cross the midline of the body [2]. Variability in clinical presentation, such as the involvement of atypical dermatomes, can affect the time and accuracy of diagnosis. A clinico-epidemiological study of herpes zoster from 2016 stated that 8.3% of patients from a cohort of 84 cases had lumbar dermatomes affected [3]. The herpes zoster rash is usually associated with severe neuralgic pain. Investigations are not generally required for the diagnosis of shingles. By obtaining a good medical history alongside a thorough physical examination (identifying the hallmark finding of shingles, which is a vesicular rash within a dermatomal distribution), it is possible to diagnose shingles.

According to UK statistics, the annual incidence of shingles in the UK is approximately 1.85-3.9 cases per 1,000 population [4]. The incidence is five times greater in individuals over 80 than in those under 50 [4]. Additionally, the prevalence increases in those who are immunosuppressed. Alongside increasing age and immunosuppression, other risk factors include infections, stress, certain comorbidities (e.g., asthma, chronic obstructive pulmonary disease (COPD), cardiovascular disorders, type 1 diabetes), and female sex [5].

Complications can occur following shingles, the most common being post-herpetic neuralgia (PHN). This is chronic nerve pain located in the same area as the shingles rash, even after the rash has subsided [6]. PHN is particularly more likely if there is facial involvement during shingles.

## Case presentation

A 90-year-old male presented to the ED with a presenting complaint of chest pain. He reported a one-day history of multiple episodes of chest pain, which was left-sided, non-radiating, sharp, and intermittent. He denied clamminess, sweating, fever, nausea, vomiting, and cough. He had presented to the hospital four days prior due to feeling generally unwell, alongside episodes of vomiting and fever. During his previous hospital visit, he was managed for a non-infective exacerbation of COPD (NIECOPD) in the ED with salbutamol and ipratropium nebulisers, alongside a five-day course of prednisolone. His WBC, CRP, and neutrophil count were within the normal range and were therefore not indicative of infection. He was discharged home when his symptoms subsided.

At home, he noticed a progressively worsening, unilateral, painful, and erythematous macular rash confined to his right knee over a period of four days (since his last presentation to the hospital). He reported pain confined to the same dermatome in the days leading up to the appearance of this rash. He visited his GP, who was suspicious of shingles. Neither the patient nor his wife (next of kin) could recall him experiencing a similar rash previously. The rash was confluent and confined to the L4 dermatome (Figure 1). During his admission, he reported that the initial pain over the rash had subsided, and he did not require any analgesia. The rash remained purely macular throughout the admission and did not progress to a vesicular rash.

**Figure 1:**
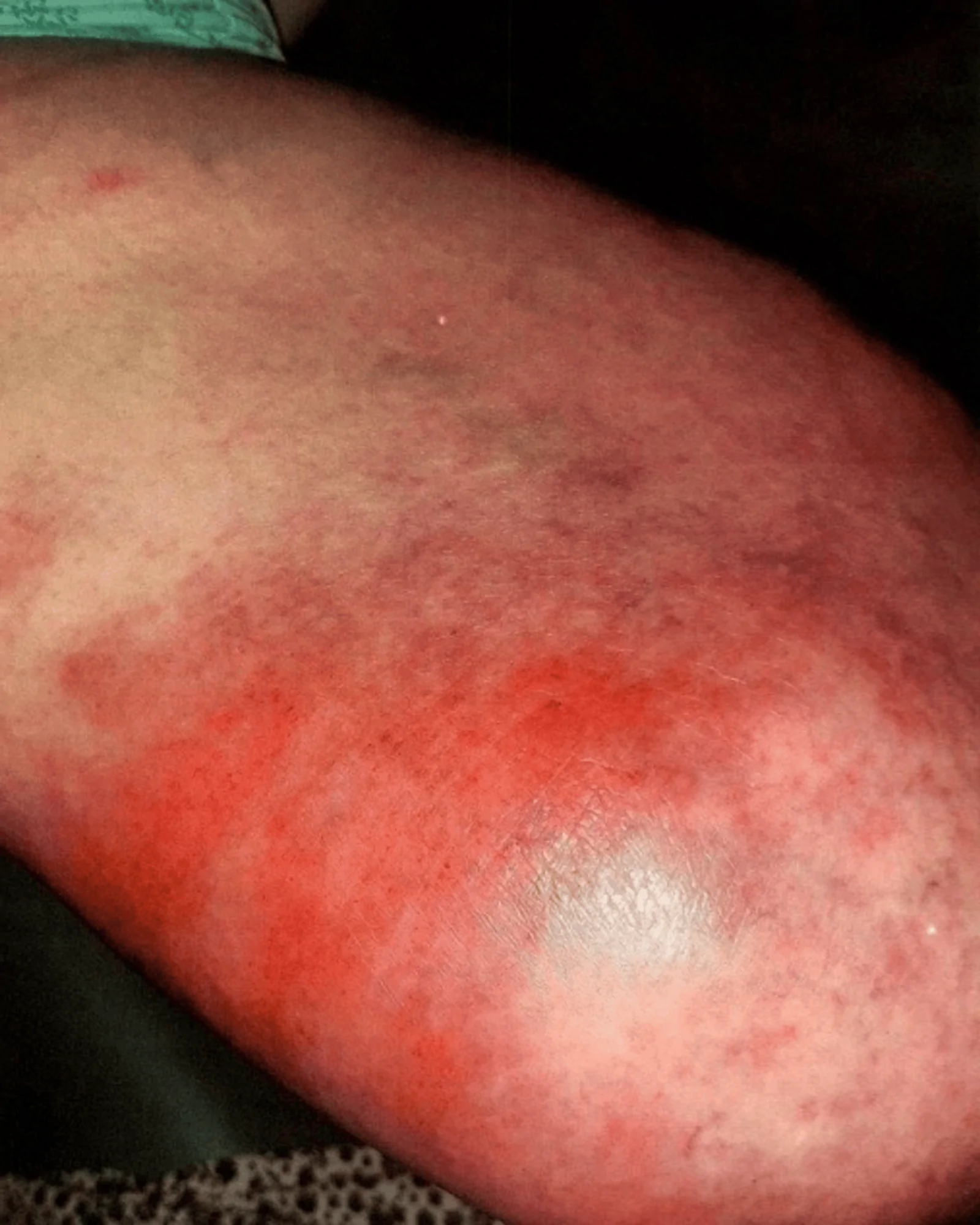
Erythematous macular rash confined to the L4 dermatome of the right leg.

This patient has an extensive past medical history, including the following: mild vascular dementia, COPD (on continuous positive airway pressure (CPAP)), osteoporosis, obstructive sleep apnoea, heart failure with reduced ejection fraction (HFrEF), chronic kidney disease (CKD) stage III, type 2 diabetes mellitus, atrial fibrillation, and pleural plaque disease due to asbestos exposure.

On physical examination, he had bilateral pedal oedema. His right leg had more pronounced swelling than the left, extending up to his shins. However, he reported that this had been ongoing for approximately two years. Important differential diagnoses to consider included cellulitis of the leg and deep vein thrombosis. He also had bibasal crackles on auscultation. His vital signs were all within the normal range. He was prescribed oral aciclovir, which he continued for a total of seven days. Considering his co-existing CKD stage III, we made sure to check his estimated glomerular filtration rate (eGFR) before prescribing aciclovir in case the dose needed to be adjusted. His eGFR was above 25 mL/min, so the normal oral dose was given: 800 mg five times a day. As mentioned in the introduction of this article, despite this being an uncommon presentation of the shingles rash, the medical team concluded that this was, in fact, an atypical presentation of shingles. This patient’s chest pain, which was present on admission, was incidental and resolved during the admission.

He was deemed medically fit after three days and was discharged home with the remainder of the aciclovir course.

## Discussion

Shingles is usually preceded by a prodromal phase lasting two to three days, but this can last for longer than seven days in some cases. Literature can be found that discusses prodromal phases lasting as long as two to three weeks [7]. During the prodromal phase, the individual may experience pruritus, paraesthesia, dysesthesia, and numbness along the affected dermatome. They may report pain in the affected dermatome of varying characteristics (e.g., burning, throbbing, shooting) and severity. The pain can be intermittent or constant [8]. Other symptoms include headaches, photophobia, malaise, and fever [9], which can steer clinicians away from including shingles in their differential diagnosis.

Following the prodromal phase, a unilateral erythematous maculopapular rash will usually appear in a dermatomal distribution. Macules are flat, discoloured, non-palpable lesions, and papules are elevated, palpable lesions. The most commonly affected dermatomes in immunocompetent individuals are T1-L2. The most common presentation of the shingles rash is the “shingles band” across the torso [10]. Multiple dermatomes can be affected in immunocompromised individuals. The associated pain can continue during this phase but can also worsen or improve. Pain may also arise for the first time during this phase. The erythematous macules and papules can develop into vesicles in 1-2 days. The formation of new vesicles can occur over 3-4 days. The vesicles can become pustular within a week of rash onset. These pustules can then ulcerate and crust after 3-5 days. The healing of these lesions can occur over 2-4 weeks. They can result in scarring and permanent discolouration [4].

Multidermatomal involvement is rare, although possible, and is more commonly associated with immunocompromised individuals. It involves multiple adjacent dermatomes [11]. Zoster duplex unilateralis or bilateralis is a herpes zoster presentation involving two noncontiguous dermatomes. The terminology depends on whether one half or both halves of the body are involved in the presentation, respectively [12].

The rash will self-resolve, although early initiation of treatment (within 72 hours of rash onset) can help to ease the pain, shorten the duration of symptoms, and reduce the risk of complications following shingles [13]. In this particular case, treatment was started within an adequate time frame, which helped to control his symptoms. Oral treatment includes aciclovir, valaciclovir, or famciclovir. These are offered to those who are immunocompromised, >50 years of age, have non-truncal involvement, have moderate to severe pain or rash, or have predisposing skin conditions.

Around 20% of individuals can experience systemic symptoms (headache, fever, malaise, and fatigue). Herpes zoster can sometimes be confused with conditions such as herpes simplex, impetigo, contact dermatitis, and scabies. A common risk factor for herpes zoster that is relevant to this patient is his advanced age (>50 years). Weaker risk factors that this patient had include COPD, CKD, cardiovascular conditions, and prior antibiotic use.

The national shingles vaccination programme began in the UK in September 2013. It initially used a single dose of the live vaccine Zostavax (Merck Sharp & Dohme LLC, Pennsylvania, USA), which was routinely offered to eligible older adults, with a catch-up programme for older age groups. Those aged 80 years and above were generally not eligible because of the reduced efficacy of the vaccine with increasing age. Shingrix (GlaxoSmithKline Biologicals SA, Wavre, Belgium), a non-live vaccine, was subsequently introduced for eligible individuals who could not receive a live vaccine. The shingles vaccination programme has since been expanded, with Shingrix now used for eligible individuals [5]. Immunocompetent individuals remain eligible for shingles vaccination until their 80th birthday. The patient discussed in this case is 90 years of age. He did not receive the shingles vaccine and is no longer eligible on the basis of age under the routine immunocompetent programme.

This case report highlights the significance of keeping in mind the variety of ways in which shingles can manifest. Furthermore, it emphasises the impact vaccination programmes have on the occurrence of herpes zoster in the ageing population. Another point to remember is to consider herpes zoster in those who present with localised swelling and rash, even if these are restricted to a less commonly affected dermatome. Early diagnosis and treatment are vital to reduce the risk of complications associated with herpes zoster.

## Conclusions

This case report highlights the significance of keeping in mind the variety of ways in which shingles can manifest, such as in atypical dermatomes. This expands clinicians' awareness of the clinical spectrum of VZV reactivation, aids earlier recognition, and reduces the risk of delayed diagnosis and inappropriate treatment. As the incidence of herpes zoster continues to rise with an ageing population and increasing numbers of immunocompromised patients, documentation of uncommon presentations remains valuable for both clinical practice and medical education.
